# Variability in Long COVID Definitions and Validation of Published Prevalence Rates

**DOI:** 10.1001/jamanetworkopen.2025.26506

**Published:** 2025-08-12

**Authors:** Lauren E. Wisk, Michelle L’Hommedieu, Kate Diaz Roldan, Imtiaz Ebna Mannan, Erica S. Spatz, Robert A. Weinstein, Arjun K. Venkatesh, Michael Gottlieb, Ryan Huebinger, Kristin L. Rising, Juan Carlos C. Montoy, Kari A. Stephens, Robert M. Rodriguez, Mandy J. Hill, Kelli N. O’Laughlin, Nicole L. Gentile, Ahamed H. Idris, Shu-Xia Li, Michelle Santangelo, Efrat R. Kean, Samuel A. McDonald, Kristyn Gatling, Joann G. Elmore

**Affiliations:** 1 Division of General Internal Medicine and Health Services Research, David Geffen School of Medicine at the University of California, Los Angeles; 2 Codetta Bio Inc, Morrisville, North Carolina; 3 Center for Outcomes Research and Evaluation (CORE), Section of Cardiovascular Medicine, Department of Internal Medicine, Yale School of Medicine, New Haven, Connecticut; 4 Department of Epidemiology, Yale School of Public Health, New Haven, Connecticut; 5 Division of Infectious Diseases, Department of Internal Medicine, Rush University Medical Center, Chicago, Illinois; 6 Division of Infectious Diseases, Department of Medicine, Cook County Hospital, Chicago, Illinois; 7 Department of Emergency Medicine, Yale School of Medicine, New Haven, Connecticut; 8 Department of Emergency Medicine, Rush University Medical Center, Chicago, Illinois; 9 Department of Emergency Medicine, UTHealth Houston, Houston, Texas; 10 Department of Emergency Medicine, Sidney Kimmel Medical School, Thomas Jefferson University, Philadelphia, Pennsylvania; 11 Center for Connected Care, Thomas Jefferson University, Philadelphia, Pennsylvania; 12 Department of Emergency Medicine, University of California, San Francisco; 13 Department of Family Medicine, University of Washington, Seattle; 14 Department of Biomedical Informatics and Medical Education, University of Washington, Seattle; 15 Department of Medicine, University of California Riverside School of Medicine; 16 Department of Emergency Medicine, University of Washington, Seattle; 17 Department of Global Health, University of Washington, Seattle; 18 Department of Rehabilitation Medicine, University of Washington, Seattle; 19 Department of Laboratory Medicine and Pathology, University of Washington, Seattle; 20 Department of Emergency Medicine, University of Texas Southwestern Medical Center, Dallas; 21 Clinical Informatics Center, University of Texas Southwestern Medical Center, Dallas

## Abstract

**Importance:**

Long COVID definitions vary widely, and no consensus exists on how to accurately measure its prevalence, complicating both clinical care and research.

**Objective:**

To assess long COVID prevalence using various definitions from published literature.

**Design, Setting, and Participants:**

This prospective, multicenter cohort study used data from the longitudinal Innovative Support for Patients With SARS-CoV-2 Infections Registry (INSPIRE). Participants aged 18 years or older with symptoms suggestive of COVID-19 illness at the time of their index SARS-CoV-2 test enrolled at 8 sites across the US from December 11, 2020, through August 29, 2022, with follow-up surveys collected through February 28, 2023.

**Exposure:**

Positive or negative SARS-CoV-2 test result at the time of acute symptoms.

**Main Outcomes and Measures:**

Long COVID prevalence among INSPIRE participants with a positive vs negative index SARS-CoV-2 test, based on long COVID definitions in published literature. Secondary outcomes were sensitivity and specificity of published definitions compared with self-reported long COVID.

**Results:**

A total of 4575 INSPIRE participants were included (mean [SD] age, 40.40 [14.58] years). Most were female (3013 of 4448 [67.7%]) and aged 18 to 49 years (3338 of 4541 [73.5%]). Applying 5 published definitions for long COVID yielded a prevalence that ranged from 30.84% (95% CI, 29.33%-32.40%) to 42.01% (95% CI, 40.37%-43.66%) at 3 months and 14.23% (95% CI, 13.01%-15.55%) to 21.94% (95% CI, 20.47%-23.47%) at 6 months postinfection; in the 5 comparator studies, reported prevalence of long COVID at 1 to 5 months postinfection ranged from 2.6% (≥84 days) to 47.4% (3-5 months) and at 6 or more months postinfection ranged from 10.0% (95% CI, 8.8%-11.0%) to 61.9% (6-11 months). Using participants’ self-reported long COVID as a criterion standard, existing published definitions had low-to-moderate sensitivity (up to 66.32% [95% CI, 62.59%-69.90%] at 3 months and 45.53% [95% CI, 41.51%-49.60%] at 6 months) and high specificity (up to 81.29% [95% CI, 79.32%-83.15%] at 3 months and 94.26% [95% CI, 92.98%-95.37%]) at 6 months.

**Conclusions and Relevance:**

In this cohort study, variability in long COVID prevalence across published definitions highlights the need for a standardized, validated definition to improve clinical recognition and research comparability, ultimately guiding more accurate diagnosis and treatment strategies.

## Key Points

**Question:**

What is the prevalence of long COVID in the Innovative Support for Patients With SARS-CoV-2 Infections Registry (INSPIRE) cohort based on definitions in published literature?

**Findings:**

In this prospective cohort study of 4575 INSPIRE participants, when replicating long COVID definitions used in the 5 published studies with the largest sample size of reviewed studies, the prevalence of long COVID among participants who were COVID-19 positive varied at 3 months from 32% to 42% and at 6 months from 15% to 22%.

**Meaning:**

The findings suggest that long COVID prevalence estimates vary as a function of disparate definitions used.

## Teaser

This cohort study uses varying definitions of long COVID from 5 published studies to estimate its prevalence in participants in the Innovative Support for Patients With SARS-CoV-2 Infections Registry.

## Introduction

There were more than 100 million cases of SARS-CoV-2 infection in the US during the COVID-19 pandemic, and many individuals report the continuation of symptoms or development of new symptoms after initial infection.^1^ Long COVID, a condition characterized by prolonged symptoms, has been variably defined.^2,3,4,5,6^ While major organizations, including the National Academies of Sciences, Engineering, and Medicine (NASEM)^7^ and others, have developed their own definitions of long COVID, there are discrepancies between the definitions,^8,9^ and many studies use their own definitions. The lack of consensus on a long COVID definition, including variations in the duration and timing of symptoms,^10^ is problematic and can lead to underrecognition or overrecognition by both patients and clinicians, resulting in delayed or unnecessary treatments.^11^ Additionally, the absence of a standardized definition (ie, true criterion standard) hinders the comparability of findings across studies.^8,12^

It is crucial to establish a universally accepted definition of long COVID that is informed by comprehensive research and that is both sensitive and specific. Without a validated, objective diagnostic tool to identify long COVID, most studies have relied on patients’ self-reported symptoms or those documented in the medical record to classify this condition.^13^ Long COVID prevalence rates are often higher in studies that use patients’ self-reported symptoms compared with studies using patients’ medical records.^14^ Patients’ self-reported data, or patient-reported outcomes, are often essential to the verification and understanding of a disease^15^ and may provide a better picture of patients’ actual lived experience than using documentation in the medical records. We aimed to describe the variability in long COVID definitions from published reports and to use self-reported patient data from the Innovative Support for Patients With SARS-CoV-2 Infections Registry (INSPIRE) to estimate the prevalence of long COVID in our study’s cohort using the various definitions from the literature.

## Methods

### Study Design and Data Source

This cohort study used data from INSPIRE, a multisite, prospective, longitudinal registry that enrolled adults who experienced acute illness with symptoms suggestive of COVID-19 from December 11, 2020, through August 29, 2022. Eight study sites across the US recruited and enrolled participants as described previously.^16^ Follow-up surveys were collected through February 28, 2023. Each study site’s institutional review board approved this study. All INSPIRE participants provided consent electronically for their deidentified data to be used in future studies. This study followed the Strengthening the Reporting of Observational Studies in Epidemiology (STROBE) reporting guideline.^17^

### Cohort Definition

INSPIRE included adult participants (aged ≥18 years) who were fluent in English or Spanish, had access to an internet-enabled device, and had self-reported symptoms suggestive of acute SARS-CoV-2 infection at the time of a SARS-CoV-2 test. Eligible individuals were tested for SARS-CoV-2 with a US Food and Drug Administration–approved or authorized molecular or antigen-based assay within 42 days prior to enrolling in the study.

Participants’ COVID-19 status (COVID-19 positive or COVID-19 negative) was based on their index SARS-CoV-2 test result at enrollment. If a participant had more than 1 test within 7 days of enrollment and had discordant test results, we considered the positive test to be the true result. If a participant’s test result changed after 7 days postenrollment in the study, we kept them in their initial COVID-19 status group.

### Variables

INSPIRE participants completed surveys at enrollment and every 3 months for up to 18 months after enrollment. At enrollment (baseline), participants self-reported their sociodemographic data, including age, gender (female, male, transgender or nonbinary, or other [ie, not listed, prefer not to answer, and gender nonconfirming]), race (Asian, Black, White, and multiracial or other [included American Indian or Alaska Native, Native Hawaiian or Other Pacific Islander, multiracial, or other responses listed in eAppendix 1 in Supplement 1]), ethnicity (Hispanic, Latino, or of Spanish origin or not Hispanic, Latino, or of Spanish origin), educational level, employment status, income, marital status, and health insurance status. Self-reported data on race and ethnicity were included in the analysis because these factors have been previously associated with long COVID prevalence.^18^ Surveys also included questions assessing physical and mental health, symptoms suggestive of COVID-19 (eTable 1 in Supplement 1), access to care, and work-related outcomes. Participants who completed the baseline survey and the 3-month follow-up were included in our analyses; participant inclusion and loss to follow-up are shown in eFigure 1 in Supplement 1. The 3-month and 6-month study time points were chosen because they most closely align with time of assessment of long COVID symptoms in the published literature.^5,6,19,20,21^ For additional analysis, we used the self-reported long COVID status from the final survey (“Regardless of whether you tested positive or negative for SARS-CoV-2 infection when you had COVID-like symptoms, do you think you had or currently have long COVID?”) to create a subset of the full sample.

### Literature Search

We conducted a literature search from March to October 2023. Published studies identified in PubMed and Google Scholar meeting the following criteria were considered: (1) researchers assessed participants’ self-reported symptoms after initial SARS-CoV-2 infection, (2) researchers provided the full list of symptoms assessed via participant self-report, (3) self-reported symptom data were used to calculate the percentage of participants with persistent symptoms after initial SARS-CoV-2 infection, and (4) studies were published in English (eAppendix 2 in Supplement 1). We chose to replicate the 5 published studies^5,6,19,20,21^ identified before November 2023 (eTable 2 in Supplement 1) with the largest total sample size of all the studies identified in the literature search. Examples of studies^22,23,24,25,26,27,28,29,30^ identified in our search but not included in our analyses are summarized in eTable 3 in Supplement 1.

To assess feasibility of replication, we compared the full list of symptoms assessed in each of the published studies against symptoms we assessed in the INSPIRE study at both 3 and 6 months. The INSPIRE survey included 29 symptoms on a checklist (21 COVID-19–like symptoms and 8 fatigue symptoms). Three investigators (M.L., K.D.R., I.E.M.) all independently compared the full list of symptoms assessed in each published study against the full list of symptoms assessed in the INSPIRE study and reached 100% agreement on whether the INSPIRE study assessed the same or similar symptoms. Additionally, in the INSPIRE survey, participants who self-reported experiencing other symptoms not included in our checklist survey were prompted to write in the other symptoms they experienced. The eMethods in Supplement 1 has information on recategorization of free-text responses.

### Statistical Analysis

Based on the definitions of long COVID used in the 5 comparator studies^5,6,19,20,21^ (Table 1), we recoded the available symptoms from the INSPIRE survey to closely match each published study. We then calculated the prevalence of long COVID for INSPIRE cohort participants using these 5 definitions. These definitions were applied to both the COVID-19–positive and COVID-19–negative samples in the INSPIRE cohorts at 3 months and 6 months; in all cases, prevalences and associated 95% CIs were calculated. The prevalences were then tested for equality based on a *Z* test with the Yates continuity correction applied. Variation attributable to the definition used was calculated as the range in prevalence estimates (ie, maximum minus minimum) divided by either the minimum or maximum prevalence.

**Table 1.** Long COVID Definitions From the 5 Studies Used for INSPIRE Replication

Source	Study location	Study period	Long COVID definition	Duration of symptoms (time point) used for INSPIRE replication	Symptoms required to classify participants with long COVID, No.	Total sample size, No.	Symptoms assessed per original study, No.
Jones et al,^19^ 2021	UK	August 7, 2020, to January 22, 2021	Self-diagnosed, clinician-diagnosed, or test-confirmed COVID-19 and had symptoms of COVID-19 that lasted >4 wk	≥4 wk	≥1	31 033	12
Pagen et al,^6^ 2023^a^	The Netherlands	November 2021 to January 2022	Had ≥1 of the 44 prelisted symptoms at 3 mo, thus reflecting for each participant the same period after positive test (ie, 3 mo)	3-5 mo, 6-11 mo	≥1	9797	44
Sudre et al,^20^ 2021	UK, US, and Sweden	March 24, 2020, to September 2, 2020	Symptoms that persisted >4 wk (28 d, LC28), >8 wk (56 d, LC56) or >12 wk (84 d, LC84) between onset and end	≥84 d	≥1	4182	14
Thaweethai et al,^5^ 2023	US and Puerto Rico	December 1, 2021, to April 10, 2023	Participants met the PASC score threshold (≥12 points); the symptoms (ordered by decreasing frequencies among participants with a qualifying PASC score) were PEM, fatigue, brain fog, dizziness, GI symptoms, palpitations, changes in sexual desire or capacity, loss of or change in smell or taste, thirst, chronic cough, chest pain, and abnormal movements	6 mo	≥1	9764	12
Yoo et al,^21^ 2022	US	April 2020 to February 2021	Persistent COVID-19 symptoms on the 90-d postdischarge survey (or the 60-d survey if the 90-d survey was incomplete)	≥60 d	≥1	1038	9

Abbreviations: GI, gastrointestinal; INSPIRE, Innovative Support for Patients With SARS-CoV-2 Infections Registry; LC, long COVID; PASC, postacute sequelae of SARS-CoV-2 infection; PEM, postexertional malaise.

^a^ Pagen et al presented 6 definitions of long-term symptoms, but we chose to replicate definition 6 because we also defined *long COVID* as participants having COVID-19 symptoms after 3 months.

We calculated the sensitivity and specificity of each definition by comparing it with participants’ self-reported long COVID status as the criterion standard, treating this patient-reported outcome as the reference category; this question did not distinguish between whether the respondent ever had or was currently experiencing the syndrome, and these responses were collected approximately 23 months after enrollment. The statistical analyses were conducted using SAS, version 9.4 (SAS Institute Inc), and R, version 4.2.2 (R Project for Statistical Computing).

## Results

Of 6044 INSPIRE participants (4547 [75.2%] COVID-19 positive) who completed a baseline survey, 4575 (75.7%; 3521 [77.0%] COVID-19 positive) completed a 3-month follow-up survey and were included in this study (eFigure 1 in Supplement 1). Among the 4575 included participants, the mean (SD) age was 40.40 (14.58) years. Of 4448 participants with available gender data, 3013 (67.7%) were female; 1363 (30.6%) were male; and 72 (1.6%) were transgender, nonbinary, or other. Of 4446 with available race data, 613 (13.8%) were Asian; 365 (8.2%) were Black; 3064 (68.9%) were White; and 404 (9.1%) were multiracial or other race (inclusive of 26 [0.6%] who were American Indian or Alaska Native, 24 [0.5%] Native Hawaiian or Other Pacific Islander, 242 [5.4%] multiracial, and 112 [2.5%] other). Of 4491 with available ethnicity data, 639 (14.2%) were Hispanic, Latino, or of Spanish origin and 3852 (85.8%) were not Hispanic, Latino, or of Spanish origin. Of 4541 with age data, 3338 (73.5%) were aged 18 to 49 years (Table 2). Moreover, 3897 participants (85.2%; 2986 [76.6%] COVID-19 positive) completed both a 3-month and a 6-month follow-up survey, and 3235 (83.0%) of those (2478 [76.6%] COVID-19 positive) also completed the final survey (eFigure 1 in Supplement 1).

**Table 2.** Baseline Demographic Characteristics of Participants in the INSPIRE Study

Characteristic	Participants^a^		
	COVID-19 positive (n = 3521)	COVID-19 negative (n = 1054)	Total (N = 4575)
Age at enrollment, y			
18-34	1433/3496 (41.0)	483/1045 (46.2)	1916/4541 (42.2)
35-49	1135/3496 (32.5)	287/1045 (27.5)	1422/4541 (31.3)
50-64	649/3496 (18.6)	187/1045 (17.9)	836/4541 (18.4)
≥65	279/3496 (8.0)	88/1045 (8.4)	367/4541 (8.1)
Missing	25	9	34
Gender			
Female	2291/3428 (66.8)	722/1020 (70.8)	3013/4448 (67.7)
Male	1092/3428 (31.9)	271/1020 (26.6)	1363/4448 (30.6)
Transgender, nonbinary, or other^b^	45/3428 (1.3)	27/1020 (2.6)	72/4448 (1.6)
Missing	93	34	127
Race^c^			
Asian	452/3431 (13.2)	161/1015 (15.9)	613/4446 (13.8)
Black	232/3431 (6.8)	133/1015 (13.1)	365/4446 (8.2)
White	2434/3431 (70.9)	630/1015 (62.1)	3064/4446 (68.9)
Multiracial or other	313/3431 (9.1)	91/1015 (9.0)	404/4446 (9.1)
Multiracial	182/3431 (5.3)	60/1015 (5.9)	242/4446 (5.4)
American Indian or Alaska Native	21/3431 (0.6)	5/1015 (0.5)	26/4446 (0.6)
Native Hawaiian or Other Pacific Islander	20/3431 (0.6)	4/1015 (0.4)	24/4446 (0.5)
Other^d^	90/3431 (2.6)	22/1015 (2.2)	112/4446 (2.5)
Missing	90	39	129
Ethnicity			
Hispanic, Latino, or of Spanish origin	465/3459 (13.4)	174/1032 (16.9)	639/4491 (14.2)
Not Hispanic, Latino, or of Spanish origin	2994/3459 (86.6)	858/1032 (83.1)	3852/4491 (85.8)
Missing	62	22	84
Educational level			
<High school	28/3447 (0.8)	16/1022 (1.6)	44/4469 (1.0)
High school graduate	197/3447 (5.7)	111/1022 (10.9)	308/4469 (6.9)
Some college	451/3447 (13.1)	196/1022 (19.2)	647/4469 (14.5)
2-y Degree	229/3447 (6.6)	82/1022 (8.0)	311/4469 (7.0)
4-y Degree	1179/3447 (34.2)	255/1022 (25.0)	1434/4469 (32.1)
>4 y of College	1363/3447 (39.5)	362/1022 (35.4)	1725/4469 (38.6)
Missing	74	32	106
Annual family income prepandemic, $			
<10 000	166 (4.7)	99 (9.4)	265 (5.8)
10 000-34 999	346 (9.8)	143 (13.6)	489 (10.7)
35 000-49 999	327 (9.3)	143 (13.6)	470 (10.3)
50 000-74 999	450 (12.8)	143 (13.6)	593 (13.0)
≥75 000	2008 (57.0)	428 (40.6)	2436 (53.2)
Unknown	224 (6.4)	98 (9.3)	322 (7.0)
Vaccination status^e^			
No	658/2773 (23.7)	155/842 (18.4)	813/3615 (22.5)
Yes	2115/2773 (76.3)	687/842 (81.6)	2802/3615 (77.5)
Missing	748	212	960
Self-reported long COVID status^f^			
No	1641/2309 (71.1)	529/691 (76.6)	2170/3000 (72.3)
Yes	668/2309 (28.9)	162/691 (23.4)	830/3000 (27.7)
Missing	1212	363	1575

Abbreviation: INSPIRE, Innovative Support for Patients With SARS-CoV-2 Infections Registry.

^a^ Data are presented as number (percentage) or number out of total number (percentage) of participants.

^b^ Other included not listed, prefer not to answer, and gender nonconfirming.

^c^ Participants could select multiple race options on the survey.

^d^ This race subcategory included responses entered manually by INSPIRE participants for “some other race.” eAppendix 1 in Supplement 1 includes a list of “some other race” responses entered.

^e^ COVID-19 vaccination indicates participants with at least 1 dose before the index SARS-CoV-2 test.

^f^ Self-reported long COVID status was an item in the final survey where participants could respond with any of the following options: (1) “no,” (2) “yes,” or (3) “I don’t know/am unsure.” We treated “I don’t know/am unsure” as a missing response in this analysis; 375 participants (10.7%) from the COVID-19 positive and 125 (11.9%) from the COVID-19 negative groups responded “I don’t know/am unsure.”

The 5 comparator studies^5,6,19,20,21^ each used distinct long COVID definitions, and the studies were conducted in different periods (Table 1 and eFigure 2 in Supplement 1). The number of symptoms from comparator studies that were matched to INSPIRE symptoms varied from 8 to 34 (Thaweethai et al,^5^ 8; Yoo et al,^21^ 9; Jones et al,^19^ 12; Sudre et al,^20^ 12; Pagen et al,^6^ 34), with some potential for one-to-many (vs one-to-one) matching when symptom categories were broadly specified. There were 5 symptoms out of the 29 from the INSPIRE questionnaire (21 COVID-19–like symptoms and 8 fatigue symptoms) that were present in all 5 of the comparator studies (“more tired than usual,” “pain or tightness in your chest,” “diarrhea [>3 loose or looser than normal stools in 24 hours],” “decreased smell or change in smell,” and “fatigue, tiredness, or exhaustion”) (eTable 4 in Supplement 1).

Of the 5 comparator studies, reported prevalence of long COVID at 1 to 5 months postinfection ranged from 2.6% (≥84 days [Sudre et al^20^]) to 47.4% (3-5 months [Pagen et al^6^]) and at 6 or more months ranged from 10.0% (95% CI, 8.8%-11.0% [Thaweethai et al^5^]) to 61.9% (6-11 months [Pagen et al^6^]). Applying these studies’ long COVID definitions to our INSPIRE cohort yielded a 3-month prevalence of long COVID that ranged from 30.84% (95% CI, 29.33%-32.40%) to 42.01% (95% CI, 40.37%-43.66%) among the COVID-19–positive group and from 28.08% (95% CI, 25.41%-30.92%) to 40.32% (95% CI, 37.35%-43.36%) among the COVID-19–negative group (Figure). We attributed 24.8% to 32.9% of the variation in long COVID prevalence to differences in the definitions used. Similarly, we calculated a 6-month prevalence of long COVID that ranged from 14.23% (95% CI, 13.01%-15.55%) to 21.94% (95% CI, 20.47%-23.47%) among the COVID-19–positive group and from 14.60% (95% CI, 12.40%-17.10%) to 23.27% (95% CI, 20.59%-26.18%) among the COVID-19–negative group. Despite similar prevalence estimates for long COVID among the cohorts that were COVID-19 positive vs COVID-19 negative (eTable 5 in Supplement 1), we identified a greater absolute number of symptoms for individuals who were COVID-19 positive who met various definitions for long COVID compared with individuals who were COVID-19 negative (eg, mean [SD] long COVID symptom count of 2.96 [2.8] among those who were COVID-19 positive vs 2.36 [2.8] among those who were COVID-19 negative per the definition of long COVID by Yoo et al^21^) (eTable 6 and eFigure 3 in Supplement 1).

**Figure.**
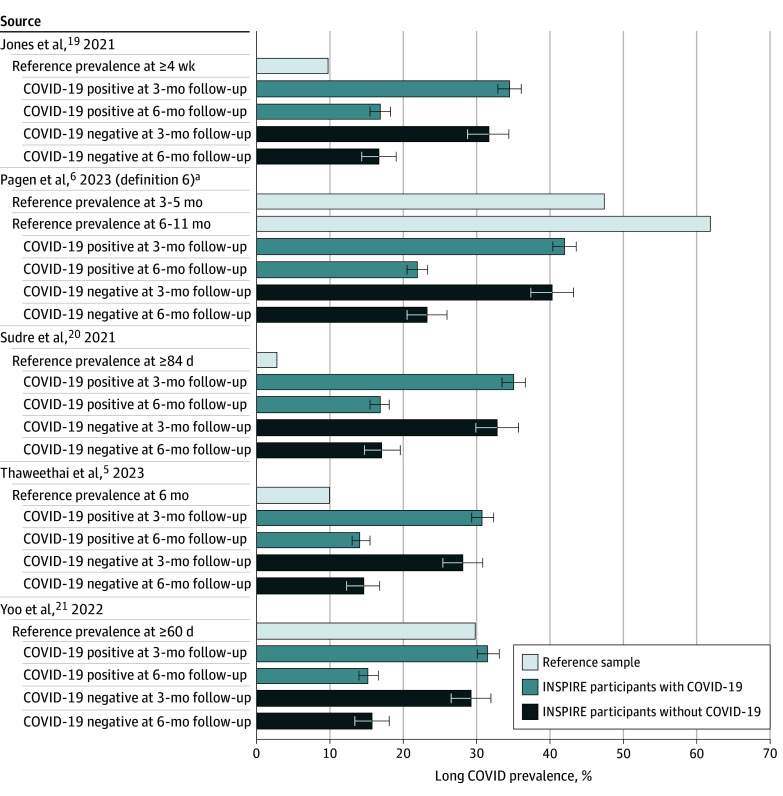
Comparison of Long COVID Prevalence Among the INSPIRE Participants When Using 5 Different Published Definitions The long COVID definition from each study was applied to eligible Innovative Support for Patients With SARS-CoV-2 Infections Registry (INSPIRE) participants who completed the surveys at 3 months and/or 6 months. The INSPIRE cohort eligible for the 3-month follow-up survey had 4575 participants and for both the 3- and 6-month surveys, 3897 participants. Prevalences were calculated based on persistence of 1 or more symptoms (constituting source definitions) at baseline and 3 months (for 3-month prevalence) and at baseline, 3 months, and 6 months (for 6-month prevalence). Error bars indicate 95% CIs. ^a^Pagen et al^6^ presented 6 definitions of long-term symptoms, but we chose to replicate definition 6 because we also defined *long COVID* as participants having COVID-19 symptoms after 3 months.

Considering the INSPIRE participants’ self-reported long COVID status as the reference category, our analyses revealed that the highest sensitivity for this patient-reported outcome was observed with the definition by Pagen et al^6^ (66.32% [95% CI, 62.59%-69.90%] at 3 months; 45.53% [95% CI, 41.51%-49.60%] at 6 months), and the highest specificity was observed with the definitions by Yoo et al^21^ (81.29% [95% CI, 79.32%-83.15%] at 3 months) and Thaweethai et al^5^ (94.26% [95% CI, 92.98%-95.37%] at 6 months) among the cohort that was COVID-19 positive (Table 3 and eTable 7 in Supplement 1). Overall, both the sensitivity and specificity of all assessed definitions were low to moderate, with sensitivity lower than specificity for all definitions.

**Table 3.** Sensitivity and Specificity When Applying Definitions of Long COVID Used in the 5 Studies to the INSPIRE Participants Who Were COVID-19 Positive in the Index Test^a^

Source study for long COVID definition	3-mo Follow-up		6-mo Follow-up	
	Sensitivity, % (95% CI)	Specificity, % (95% CI)	Sensitivity, % (95% CI)	Specificity, % (95% CI)
Jones et al,^19^ 2021	59.28 (55.45-63.03)	78.43 (76.36-80.40)	39.07 (35.16-43.09)	93.02 (91.63-94.24)
Pagen et al,^6^ 2023	66.32 (62.59-69.90)	70.57 (68.30-72.76)	45.53 (41.51-49.60)	88.52 (86.82-90.07)
Sudre et al,^20^ 2021	59.13 (55.30-62.89)	77.27 (75.16-79.28)	39.07 (35.16-43.09)	93.09 (91.70-94.31)
Thaweethai et al,^5^ 2023	54.04 (50.18-57.87)	81.05 (79.07-82.92)	31.79 (28.09-35.67)	94.26 (92.98-95.37)
Yoo et al,^21^ 2022	55.54 (51.68-59.35)	81.29 (79.32-83.15)	35.26 (31.45-39.22)	93.93 (92.62-95.08)

Abbreviation: INSPIRE, Innovative Support for Patients With SARS-CoV-2 Infections Registry.

^a^ The sensitivity and specificity of prevalence estimates were calculated by considering as the criterion standard the self-reported response of INSPIRE participants who tested positive for COVID-19 in the index test to the final survey (which asked, “Regardless of whether you tested positive or negative for SARS-CoV-2 infection when you had COVID-like symptoms, do you think you had or currently have long COVID?”). Six-month results indicate results from participants who completed both 3- and 6-month surveys.

## Discussion

We found that researchers’ decision to use any of a range of published long COVID definitions could result in considerable variation in the prevalence estimate obtained from their cohort. Furthermore, we identified that most definitions had less than optimal sensitivity and specificity for identifying subjectively reported long COVID, indicating that standard definitions do not align with patient-reported experience of long COVID. In addition, we found that the most relevant distinguishing feature between potential long COVID cases (ie, among COVID-19–positive cases) vs other postillness conditions (ie, among COVID-19–negative cases) was the overall symptom count, with more symptoms present in the long COVID cases.

NASEM released a 2024 long COVID definition and encourages the federal government, clinicians, and others to adopt this new definition to aid in consistent diagnosis, documentation, and treatment.^7^ As the report rightfully acknowledges, the lack of consensus on the definition of long COVID hinders accurate comparison across long COVID studies^9^ and may lead to challenges in identification and evaluation of subsequent treatment.^11^ The suggested NASEM definition, however, is extremely broad (presence of any of hundreds of symptoms for at least 3 months as a continuous or relapsing and remitting symptom) and does not require laboratory confirmation or other proof of the initial infection.^7^ As our study only assessed 29 checklist symptoms with an “other” (free-text) option (substantially fewer symptoms than NASEM suggests), we were not able to report results using that operationalization. Given the broad NASEM definition, its specificity will likely be low with substantial false-positive results.^31^

Differences in the list of symptoms used for defining long COVID accounted for 24.8% to 32.9% of the variation in published prevalence rates among the 5 studies we assessed. The long COVID definitions from the published literature that we evaluated included from 9^21^ up to 44^6^ total symptoms, whereas the NASEM definition is far broader^31^ and is likely to lead to increased prevalence and improved sensitivity at the expense of misclassification from increased false-positives. While this may be considered a sensible tradeoff given that we found that the evaluated definitions generally performed better in terms of specificity rather than sensitivity, the goals of such a decision need to be carefully considered to determine which test characteristic should be maximized. A large number of symptoms to assess may overwhelm clinicians; thus, the ease of use of the definition in clinical practice should also be considered. Based on experiences using lists of symptoms for diagnosis of other diseases without clear-cut biologic markers, like Behçet or Still disease,^32^ the lists are more easily used in clinical practice when they are parsimonious and targeted or if there are clear clinical markers (which is not the case with long COVID^33^). If invasive interventions are developed for treating long COVID, greater specificity will be important to avoid overtreatment as well as inappropriately labeling individuals with a diagnosis that may affect their approach to life.

Of note, the most distinguishing feature of INSPIRE participants who were COVID-19 positive and were identified as having long COVID was not simply the presence of symptoms at 3 months postinfection but the number of symptoms present; individuals who were COVID-19 positive tended to have a greater mean number of reported symptoms than did their counterparts who were COVID-19 negative but who otherwise would have met the definition for long COVID. Though we did not compare severity or functional impairment (consistent with the 2024 NASEM recommendations), modifying definitions to include a minimum number of discrete symptoms may improve the ability of such an algorithm to uniquely identify long COVID among those with a history of COVID-19. The long COVID definition will undoubtedly evolve over time as new evidence and understanding emerge.

In addition, our finding that up to one-third of the variation in reported prevalence of long COVID was likely due to the varying definitions in use suggests that the remaining two-thirds of variation may be due to differences in sample characteristics (eg, higher prevalence rates observed in studies of hospitalized patients compared with patients recruited from the general population) or potentially due to the timing of the study (Figure) and, thus, due to different SARS-CoV-2 variants or availability of vaccines.^34^ Future work is needed to fully evaluate the extent to which certain patient characteristics, variants, and vaccination are responsible for making some subpopulations susceptible to experiencing long COVID.^35^ This will be critically important for addressing equity issues in the diagnosis and treatment of long COVID moving forward.

### Strengths and Limitations

Our study has several strengths. First, we included a comparison group comprising participants who were symptomatic but tested negative for SARS-CoV-2.^36^ This comparison group allowed us to calculate rates of long COVID in a cohort that was COVID-19 negative, thus providing data on potential misclassification when using these definitions in a cohort without a confirmed positive SARS-CoV-2 test—especially relevant given that the 2024 NASEM^7^ definition does not require evidence of positive testing for SARS-CoV-2. We additionally evaluated the long COVID prevalence using a shorter time frame for assessment (3 months, as suggested by NASEM^7^) vs longer (6 months), highlighting not only the extent to which prevalence rates decline over time but also how the ability of various definitions for distinguishing long COVID from other postacute syndromes declines as well. In addition, our use of a geographically diverse cohort with representation across several COVID-19 variants (ie, pre-Delta, Delta, and Omicron) supports the generalizability of these findings regarding variant.

Our study also has several limitations. First, participants may be subject to recall bias when self-reporting symptoms,^37^ and we did not examine participants’ electronic health records to see if symptoms were also documented in their medical records. However, some symptoms, such as fatigue, impaired smell and taste, and chest pain, are more likely to be reported in self-reported surveys than in health care records.^38^ Patients’ self-reported symptoms still provide important insights into how patients’ daily activities and functioning are affected,^38^ and their use may mitigate potential biases introduced by incomplete or inconsistent documentation in a medical record.^39^ Second, we did not exclude patients (either COVID-19 positive or COVID-19 negative) who had a SARS-CoV-2 infection after their baseline survey; thus, we may have miscalculated the prevalence of long COVID after a single infection. Similarly, our measure of self-reported long COVID included the experience of this condition at any point up to 23 months after the initial infection and so may lead to misestimation of self-reported long COVID after a single infection. Third, we did not examine whether participant demographics, such as race and ethnicity, were associated with prevalence estimates for each long COVID definition, though prior work by our group has identified similar symptom reporting across racial and ethnic groups.^40^ Fourth, we only used the long COVID definitions from 5 published studies; results may have varied if we used different definitions from other published studies. Moreover, we did not achieve perfect alignment of symptoms assessed within our cohort and the 5 studies we evaluated, which may have led to an underestimation of prevalence in our sample.

## Conclusions

In this cohort study of long COVID prevalence, variability in prevalence across 5 different published definitions highlighted the need for a standardized, validated definition to improve clinical recognition and research comparability, ultimately guiding more accurate diagnosis and treatment strategies. We described significant variation in reported long COVID definitions and prevalence rates and provided data on sensitivity and specificity of these different definitions in the study’s INSPIRE cohort. Some definitions outperformed others regarding uniquely identifying patient-reported long COVID in a sample that was COVID-19 positive compared with COVID-19 negative, but differences were generally small. Future epidemiologic investigations into long COVID should seek to apply a consistent epidemiologic definition, which likely will differ from a broader clinical definition because of the subjective nature of many long COVID symptoms and the broader uses of clinical definitions (eg, for insurance decisions and reimbursement). A more parsimonious list of symptoms for defining long COVID epidemiologically, or requiring that a minimum of 2 symptoms be reported, may add needed specificity, which is important for informing which patients to include in intervention trials and in studies of pathogenesis. Further definition refinements ideally would be based on an understanding of the pathophysiologic mechanisms underlying long COVID; without such insights, a single definition for clinical and epidemiologic use (ie, for research and public health) is unlikely.

## Notes

Supplement 1. **eAppendix 1**. List of “Other” Races Entered by INSPIRE Participants **eTable 1.** Symptoms Assessed in the INSPIRE Survey **eFigure 1.** INSPIRE Participant Flow Diagram **eAppendix 2.** Search Criteria Used in PubMed and Google Scholar **eTable 2.** Long COVID Prevalence Rate Studies Used for INSPIRE Replication **eTable 3.** A Summary of 9 Published Studies That We Did Not Replicate **eMethods.** Our Methods for Recoding INSPIRE Participants’ “Other” Symptoms **eFigure 2.** Timeline of Data Collection for Studies Used for INSPIRE Replication **eTable 4.** Symptoms Assessed in the INSPIRE Study Compared to the Five Studies **eTable 5.** Comparison of Long COVID Prevalence From the Studies With INSPIRE COVID Cohorts **eTable 6.** Symptom Count by Long COVID Definition **eFigure 3.** Mean Symptom Count by Long COVID Definition at 3 Months and 6 Months **eTable 7.** 2 × 2 Tables Comparing Long COVID Counts From the INSPIRE Final Survey (Participant Self-Report) vs Counts After Applying the Long COVID From 5 Studies
eReferences

Supplement 2.
INSPIRE Group members

Supplement 3.
Data Sharing Statement
