# Supplementary material for: Variability in Long COVID Definitions and Validation of Published Prevalence Rates
Source: JAMA Netw Open. 2025 Aug 12;8(8):e2526506. doi: 10.1001/jamanetworkopen.2025.26506 (PMC12344537; doi:10.1001/jamanetworkopen.2025.26506)
Supplement: Supplement 1. — eAppendix 1. List of “Other” Races Entered by INSPIRE Participants eTable 1. Symptoms Assessed in the INSPIRE Survey eFigure 1. INSPIRE Participant Flow Diagram eAppendix 2. Search Criteria Used in PubMed and Google Scholar eTable 2. Long COVID Prevalence Rate Studies Used for INSPIRE Replication eTable 3. A Summary of 9 Published Studies That We Did Not Replicate eMethods. Our Methods for Recoding INSPIRE Participants’ “Other” Symptoms eFigure 2. Timeline of Data Collection for Studies Used for INSPIRE Replication eTable 4. Symptoms Assessed in the INSPIRE Study Compared to the Five Studies eTable 5. Comparison of Long COVID Prevalence From the Studies With INSPIRE COVID Cohorts eTable 6. Symptom Count by Long COVID Definition eFigure 3. Mean Symptom Count by Long COVID Definition at 3 Months and 6 Months eTable 7. 2 × 2 Tables Comparing Long COVID Counts From the INSPIRE Final Survey (Participant Self-Report) vs Counts After Applying the Long COVID From 5 Studies eReferences [file jamanetwopen-e2526506-s001.pdf]

# Supplementary Online Content

Wisk LE, L'Hommedieu M, Diaz Roldan K, et al; for the INSPIRE Group. Variability in long COVID definitions and validation of published prevalence rates. *JAMA Netw Open*. 2025;8(8):e2526506. doi:10.1001/jamanetworkopen.2025.26506

**eAppendix 1.** List of "Other" Races Entered by INSPIRE Participants

**eTable 1.** Symptoms Assessed in the INSPIRE Survey

**eFigure 1.** INSPIRE Participant Flow Diagram

**eAppendix 2.** Search Criteria Used in PubMed and Google Scholar

**eTable 2.** Long COVID Prevalence Rate Studies Used for INSPIRE Replication

**eTable 3.** A Summary of 9 Published Studies That We Did Not Replicate

**eMethods.** Our Methods for Recoding INSPIRE Participants' "Other" Symptoms

**eFigure 2.** Timeline of Data Collection for Studies Used for INSPIRE Replication

**eTable 4.** Symptoms Assessed in the INSPIRE Study Compared to the Five Studies

**eTable 5.** Comparison of Long COVID Prevalence From the Studies With INSPIRE COVID Cohorts

**eTable 6.** Symptom Count by Long COVID Definition

**eFigure 3.** Mean Symptom Count by Long COVID Definition at 3 Months and 6 Months

**eTable 7.** 2 × 2 Tables Comparing Long COVID Counts From the INSPIRE Final Survey (Participant Self-Report) vs Counts After Applying the Long COVID From 5 Studies

**eReferences**

This supplementary material has been provided by the authors to give readers additional information about their work.

© 2025 Wisk LE et al. *JAMA Network Open*.

## eAppendix 1. List of “Other” races entered by INSPIRE participants

| **Write in responses:** |
|---|
| 3 or more Eastern European countries |
| 51 percent European/39 percent Native American genes. |
| American |
| Ashkenazi Jewish |
| Bi Racial |
| Blend of Black,Spanish and Native |
| Brown |
| Carribean or Black |
| Chaldean |
| Eg |
| Filipino |
| Hispana Mexicana |
| Hispanic |
| Hispanic/Latina |
| Indian Subcontinent |
| Iran |
| Jewish |
| Latin |
| Latin0 |
| Latina |
| Latino |
| Latino/ Hispanic |
| Latinx |
| Lebanese |

© 2025 Wisk LE et al. *JAMA Network Open*.

| **Write in responses:** |
|---|
| MENA |
| Mediterranean |
| Mestizo |
| Mexican |
| Mexican American |
| Mexican and middle eastern |
| Mexican/American |
| MexiffdD1 |
| Middle Eastern |
| Middle Eastern/ Egyptian |
| Middle eastern |
| Middle-eastern |
| Mix |
| Mixed |
| Multi |
| Native American |
| Native American/European |
| Native/Spaniard/African |
| North Frican |
| Not white |
| Other |
| Prefer not to answer |
| Puerto Rican |
| Southamerican Latino |
| Spanish Origin |

© 2025 Wisk LE et al. *JAMA Network Open*.

| Write in responses: |
| --- |
| Unknown kind |
| West Indian |
| hispanic |
| italian |
| latino |
| mestiza |
| middle eastern |

© 2025 Wisk LE et al. *JAMA Network Open*.

## eTable 1. Symptoms assessed in the INSPIRE survey

| INSPIRE Survey Symptoms |
|---|
| **Symptom assessment section of the INSPIRE survey**<br>*Since you first felt sick with COVID-19 like symptoms, have you had any of the following (select yes for each)?* |
| a. Fever >100.4F (38C)? |
| b. Feeling hot or feverish? |
| c. Chills? |
| d. Repeated shaking with chills? |
| e. More tired than usual? |
| f. Muscle aches? |
| g. Joint pains? |
| h. Runny nose |
| i. Sore throat? |
| j. A new cough, or worsening of a chronic cough? |
| k. Shortness of breath? |
| l. Wheezing? |
| m. Pain or tightness in your chest? |
| n. Palpitations? |
| o. Nausea or vomiting? |
| p. Headache? |
| q. Hair loss? |
| r. Abdominal pain? |
| s. Diarrhea (>3 loose/looser than normal stools/24 hours)? |
| t. Decreased smell or change in smell? |
| u. Decreased taste or change in taste? |
| v. Other (insert, specify which other symptoms as a write in text option) |
| **Fatigue symptoms questions section of the INSPIRE survey**<br>*Below is a list of symptoms many individuals experience. Please tell us whether or not you have any of the symptoms listed below.* |
| a. Fatigue, tiredness, or exhaustion |
| b. Muscle aches/muscle pains |
| c. Pain in joints |
| d. Unrefreshing sleep |
| e. Problems getting to sleep, sleeping through the night, or waking up on time |
| f. Forgetfulness/memory problems that caused you to substantially cut back on your activities |
| g. Difficulty thinking or concentrating that caused you to substantially cut back on your activities |
| h. Dizziness or fainting |

© 2025 Wisk LE et al. *JAMA Network Open*.

## eFigure 1. INSPIRE participant flow diagram

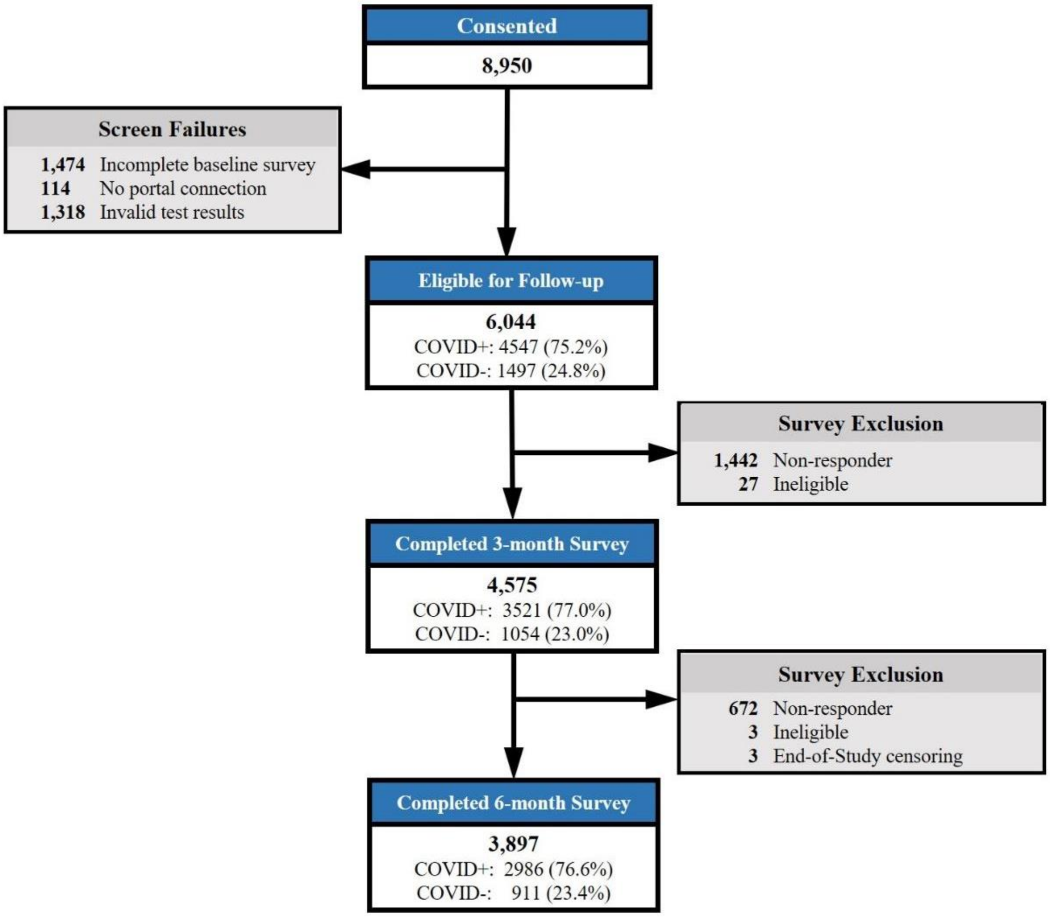


Portal connection was requirement for follow-up eligibility from study start through 3/21/22.
Invalid covid test results = no proof of test or had a positive test >42 days ago.
For sensitivity and specificity calculation, Long COVID responses (survey question: “Regardless of whether you tested positive or negative for SARS-CoV-2 infection when you had COVID-like symptoms, do you think you had or currently have Long COVID ?”; responses: “No”, “Yes”, “I don’t know/ am unsure.”) from the final INSPIRE survey (N=3498) were used.

© 2025 Wisk LE et al. *JAMA Network Open*.

## eAppendix 2. Search criteria used in PubMed and Google Scholar

We searched terms such as “Long COVID”, “definitions”, “post-COVID-19 syndrome”, “long covid prevalence”, “definition of long covid”, and “persistent symptoms of covid-19” in PubMed and Google Scholar to identify studies.

© 2025 Wisk LE et al. *JAMA Network Open*.

## eTable 2. Long COVID prevalence rate studies used for INSPIRE replication

| Last name of first author/Year | Dates of data collection | Study Location | Time points assessed | Total sample size | Age Range of Total Sample | Race/Ethnicity of Total Sample | % of participants with Long COVID | Control Group | Hospitalization Data | Number of symptoms assessed |
|---|---|---|---|---|---|---|---|---|---|---|
| Jones et al.,[1,a] 2021 | August 7th, 2020 to January 22nd, 2021 | United Kingdom | Authors classified Long COVID at >4 weeks (for main study) and >12 weeks (for sensitivity analysis) | n = 31,033 | ≥ 18 years | **White:**<br>96.2% (278 out of 310 participants with Long COVID)<br><br>91.8% (2,162 out of 2,841 participants whose COVID-19 symptoms lasted for <4 weeks)<br><br>**Non-White:**<br>3.8% (11 out of 310 participants with Long COVID)<br><br>8.2% (194 out of 2,841 participants whose COVID-19 symptoms lasted for <4 weeks) | 9.8% (310 out of 3,151) of COVID-19 participants had Long COVID | No | 28 out of 310 (9.0%) participants with Long COVID were admitted to the hospital.<br><br>54 out of 2,841 (1.9%) participants, whose COVID-19 symptoms lasted for <4 weeks, were admitted to the hospital | 12 |
| Pagen et al.,[2,b] 2023 | November 2021 and January 2022 | Netherlands | Authors classified long-term symptoms at 3 months after testing for SARS-CoV-2 | n = 9,797 | ≥ 18 years | None | Long-term symptoms prevalence among COVID+ participants ranged between 47.4% and 64.1% at 3 months. | Yes | During infection, 99 out of 7,405 (1.3%) COVID+ participants | 44 |

© 2025 Wisk LE et al. *JAMA Network Open*.

| | | | | | | | | | | |
|---|---|---|---|---|---|---|---|---|---|---|
| | | | | | | | Long-term symptoms prevalence among participants who tested positive 3-5 months ago was 47.4%.<br>Long-term symptoms prevalence among participants who tested positive 6-11 months ago was 61.9%.<br>Long-term symptoms prevalence among participants who tested positive ≥12 months ago was 64.1% | | were hospitalized. No information about the hospitalization of the full cohort. | |
| Sudre et al.,[3] 2021 | March 24th and September 2nd, 2020 | United Kingdom, United States and Sweden | Participants experienced symptoms that lasted ≥ 28, ≥ 56, and ≥ 84 days | n = 4,182 | ≥ 18 years | None | 13.3% (558 out of 4,182) of COVID+ participants still experienced symptoms that lasted ≥ 28 days<br>4.5% (189 out of 4,182) of COVID+ participants still experienced symptoms that lasted ≥ 56 days<br>2.6% (108 out of 4,182) of COVID+ participants still experienced symptoms that lasted ≥ 84 days | Yes | COVID+ participants' visit to hospital =<br>13.9%<br><br>COVID- participants' visit to hospital = 4.1% | 14 |

© 2025 Wisk LE et al. *JAMA Network Open*.

| | | | | | | | | | | |
|---|---|---|---|---|---|---|---|---|---|---|
| Thaweethai et al.,[4,c] 2023 | December 1st, 2021 - April 10th, 2023 | United States and Puerto Rico | For analysis, authors used the first visit at ≥ 6 months after the first SARS-CoV-2 test result date or date of the onset of symptoms | n = 9,764 | ≥ 18 years | **Asian, non-Hispanic:**<br>5% (428 out of 8,558 of infected participants)<br><br>7% (73 out of 1,106 of uninfected participants)<br>**Black or African American, non-Hispanic:**<br>14% (1,220 out of 8,558 of infected participants)<br><br>18% (197 out of 1,106 of uninfected participants)<br>**Hispanic, Latino or Spanish:**<br>17% (1,473 out of 8,558 of infected participants)<br><br>11% (119 out of 1,106 of uninfected participants)<br>**White, non-Hispanic:**<br>59% (5,027 out of 8,558 of infected participants)<br><br>62% (685 out of 1,106 of | 10.0% (224 out of 2231) of infected participants had Long COVID at 6 months. | Yes | No information about the hospitalization of the full cohort. | 12 |

© 2025 Wisk LE et al. *JAMA Network Open*.

| | | | | | | | | | | |
|---|---|---|---|---|---|---|---|---|---|---|
| | | | | | | uninfected participants)<br>**Multiracial/multiethnic:**<br>4% (305 out of 8,558 of infected participants)<br><br>2% (26 out of 1,106 of uninfected participants)<br>**Other:**<br>1% (105 out of 8,558 of infected participants)<br><br>1% (6 out of 1,106 of uninfected participants) | | | | |
| Yoo et al.,[5,d] 2022 | April 2020 - February 2021 | United States | Authors followed up participants over time at 30, 60, and 90 days after their discharge from the hospital or outpatient diagnosis<br><br>Participants still experienced symptoms at 60-90 days after COVID-19 illness | n = 1,038 | None | **White:** 30.5% (317 out of 1,038)<br><br>**Hispanic or Latino:** 41.6% (432 out of 1,038)<br><br>**Black:** 7.1% (74 out of 1,038)<br>**Asian:** 7.9% (82 out of 1,038)<br>**Other race:** 8.2% (85 out of 1,038)<br>**Unknown:** 4.6% (48 out of 1,038) | 29.8% (309 out of 1,038) of participants still experienced symptoms at 60-90 days after infection | No | 800 out of 1,038 (77.1%) participants were hospitalized | 9 |

**Notes:**
[a]Authors only presented race/ethnicity information for their 3,151 COVID-19 participants.
[b]Authors presented 6 definitions of long-term symptoms, but we chose to replicate definition 6 (≥1 of the 44 prelisted symptoms at 3 months, thus reflecting for each participant the same time period after positive test (i.e. 3 months)) because we are also defining Long COVID as participants having COVID-19 symptoms after 3 months.

© 2025 Wisk LE et al. *JAMA Network Open*.

[c]Authors only presented race/ethnicity information for 9,664 participants.
[d]Study was conducted at UCLA, thus there may be overlap in patient population to INSPIRE study.

© 2025 Wisk LE et al. *JAMA Network Open*.

## eTable 3. A summary of 9 published studies that we did not replicate

| Last name of first author/Year | Article title | Journal | Total sample size | Number of symptoms assessed |
|---|---|---|---|---|
| Augustin et al.,[6] 2021 | Post-COVID syndrome in non-hospitalised patients with COVID-19: a longitudinal prospective cohort study. | The Lancet Regional Health – Europe | n= 958 | 12 |
| Carvalho-Schneider et al.,[7] 2021 | Follow-up of adults with noncritical COVID-19 two months after symptom onset | Clinical Microbiology and Infection | n= 150 | 13 |
| Desgranges et al.,[8] 2022 | Post-COVID-19 Syndrome in Outpatients: a Cohort Study | Journal of General Internal Medicine | n= 507 | 14 |
| Kisiel et al.,[9] 2022 | Predictors of post-COVID-19 and the impact of persistent symptoms in non-hospitalized patients 12 months after COVID-19, with a focus on work ability | Upsala Journal of Medical Sciences | n= 336 | 20 |
| Klein et al.,[10] 2021 | Onset, duration and unresolved symptoms, including smell and taste changes, in mild COVID-19 infection: a cohort study in Israeli patients | Clinical Microbiology and Infection | n= 103 | 14 |
| Lapa et al.,[11] 2023 | Prevalence and Associated Factors of Post-COVID-19 Syndrome in a Brazilian Cohort after 3 and 6 Months of Hospital Discharge | International Journal of Environmental Research and Public Health | n= 400 | 17 |
| Morioka et al.,[12,a] 2022 | Post COVID-19 condition of the Omicron variant of SARS-CoV-2 | Journal of Infection and Chemotherapy | n=555 | 10 |
| Peghin et al.,[13] 2021 | Post-COVID-19 symptoms 6 months after acute infection among hospitalized and non-hospitalized patients | Clinical Microbiology and Infection | n = 599 | 15 |
| Román-Montes et al.,[14] 2023 | Post-COVID-19 syndrome and quality of life impairment in severe COVID-19 Mexican patients | Frontiers in Public Health | n = 246 | 28 |

**Notes**: These studies were not included in our replication, as they were not the 5 studies with the largest total sample size.
[a]We presented the total sample size before propensity score matching. Total sample size after propensity score matching: n= 36

© 2025 Wisk LE et al. *JAMA Network Open*.

## eMethods. Our methods for recoding INSPIRE participants' "other" symptoms

INSPIRE participants wrote in 876 "other" free-text responses that they experienced. The research assistant (KDR) compared the multiple symptoms from the free-text responses to those assessed in each of the 5 selected papers to identify if any of our participants "other" symptoms could be recoded as any of the symptoms assessed in those studies (e.g., loss of appetite). KDR also compared INSPIRE participants' "other" symptoms to the list of symptoms assessed in the INSPIRE study to identify symptoms that could be recoded as any that were already assessed in INSPIRE (e.g., fever). If an "other" symptom had close overlap with a symptom assessed in INSPIRE or in any of the 5 papers, the 'other' symptom was recoded to the named symptom. If an "other" response was uninterpretable (e.g., "Hiatami"), it was not recoded. The project director (ML) and analyst (IEM) conducted initial, random spot-checking of KDR's assessment of "other" responses and found no areas of disagreement. Of the 876 free-text responses, we were able to recategorize these responses into 12 new (in addition to the list of 29 symptoms from the INSPIRE questionnaire) symptom categories. The table below highlights the new symptom categories (based on INSPIRE free-text data) that were matched to existing symptoms from the comparator studies. Of note, because we relied on "other" (write in) symptoms to improve alignment between symptoms assessed in our cohort and those of the papers with which we were comparing, we expect that some of these symptoms would be underreported.

| New symptom category # | Jones et al.,[1] 2021 | Pagen et al.,[2] 2023 | Sudre et al.,[3] 2021 | Thaweethai et al.,[4] 2023 | Yoo et al.,[5] 2022 |
|---|---|---|---|---|---|
| 1 | Loss of appetite | Loss of appetite | | | |
| 2 | Confusion or disorientation | Confusion | | | |
| 3 | | Brain fog | | Brain fog | |
| 4 | | Voice difficulties | Hoarse voice | | |
| 5 | | Tinnitus | | | |
| 6 | | Skin rash/red spots on toes or feet | | | Rash |
| 7 | | Eye difficulties | | | |
| 8 | | Dreariness/depression | | | |
| 9 | | | | GI symptoms | |
| 10 | | | | Abnormal movements | |
| 11 | | | | | Numbness or tingling |
| 12 | | | | | Night sweats |

© 2025 Wisk LE et al. *JAMA Network Open*.

**eFigure 2. Timeline of data collection for studies used for INSPIRE replication**

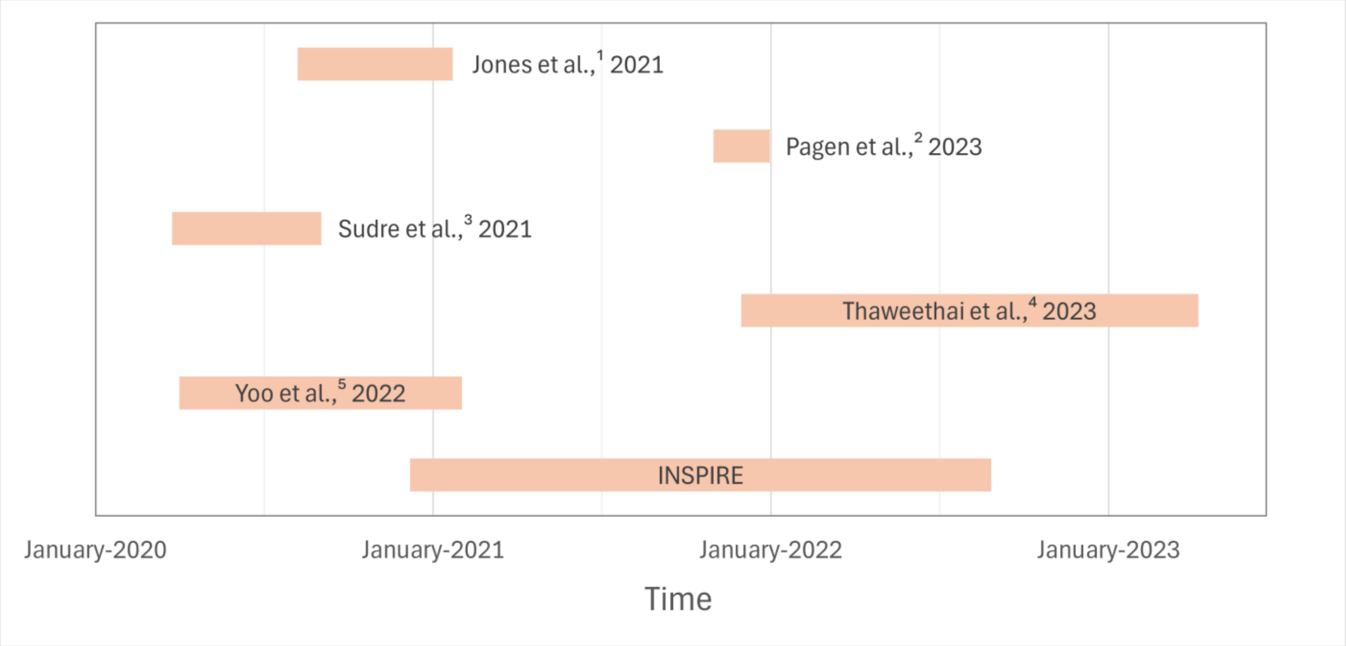


The study period for the studies are as follows:

**Jones** et al.,[1] 2021: August 07 2020 – January 22 2021
**Pagen** et al.,[2] 2023: November 01 2021 – January 01 2022
**Sudre** et al.,[3] 2021: March 24 2020 – September 02 2020
**Thaweethai** et al.,[4] 2023: December 01 2021 – April 10 2023
**Yoo** et al.,[5] 2022: April 01 2020 – February 01 2021
**INSPIRE**: December 07 2020 – August 29 2022.

Thaweethai et al. included data on persons infected before December 1, 2021, but did not specify the exact start date, so we include the date for their acute Omicron cohort, for which we report their estimates.

© 2025 Wisk LE et al. *JAMA Network Open*.

## eTable 4. Symptoms assessed in the INSPIRE study compared to the five studies

| INSPIRE | Jones et al.,[1,a] 2021 | Pagen et al.,[2,b] 2023 | Sudre et al., [3,c] 2021 | Thaweethai et al.,[4,d] 2023 | Yoo et al.,[5,e] 2022 |
|---|---|---|---|---|---|
| **Symptom assessment section of the INSPIRE survey.** *Since you first felt sick with COVID-19 like symptoms, have you had any of the following (select yes for each)?* | | | | | |
| a. Fever >100.4F (38C)? | X | X | X | | X |
| b. Feeling hot or feverish? | | X | | | |
| c. Chills? | | X | | | X |
| d. Repeated shaking with chills? | | | | | |
| e. More tired than usual? | X | X | X | X | X |
| f. Muscle aches? | X | X | X | | X |
| g. Joint pains? | X | X | | | |
| h. Runny nose | | X | | | |
| i. Sore throat? | | X | X | | |
| j. A new cough, or worsening of a chronic cough? | X | X | X | X | |
| k. Shortness of breath? | X | X | X | | X |
| l. Wheezing? | | | | | |
| m. Pain or tightness in your chest? | X | X | X | X | X |
| n. Palpitations? | | X | | X | |
| o. Nausea or vomiting? | | X | | X | X |
| p. Headache? | X | X | X | | |
| q. Hair loss? | | X | | | |
| r. Abdominal pain? | X | X | X | | |
| s. Diarrhea (>3 loose/looser than normal stools/24 hours)? | X | X | X | X | X |
| t. Decreased smell or change in smell? | X | X | X | X | X |
| u. Decreased taste or change in taste? | X | X | | X | X |
| v. Other (insert, specify which other symptoms as a write in text option) | | | | | |
| **Fatigue symptoms questions section of the INSPIRE survey.** *Below is a list of symptoms many individuals experience. Please tell us whether or not you have any of the symptoms listed below.* | | | | | |
| a. Fatigue, tiredness, or exhaustion | X | X | X | X | X |
| b. Muscle aches/muscle pains | X | X | X | | X |
| c. Pain in joints | X | X | | | |
| d. Unrefreshing sleep | | X | | | |
| e. Problems getting to sleep, sleeping through the night, or waking up on time | | X | | | |
| f. Forgetfulness/memory problems that caused you to substantially cut back on your activities | | X | | X | |

© 2025 Wisk LE et al. *JAMA Network Open*.

| | | | | | |
|---|---|---|---|---|---|
| g. Difficulty thinking or concentrating that caused you to substantially cut back on your activities | | X | | X | |
| h. Dizziness or fainting | | X | | X | |

**Notes:** "X" indicates that an INSPIRE symptom (often one-to-many) was also assessed in a comparator paper.
[a]Jones et al. reported following symptoms (n=12): abdominal pain; aches and pains; loss of appetite; chest pain; confusion or disorientation; diarrhea; fatigue or tiredness; fever or high temperature; headache; loss or change of smell or taste; persistent dry cough; shortness of breath.[1] In addition to the matches from the table (n=15), we also mapped loss of appetite and confusion or disorientation from "other" free-text symptoms (n=2). No symptoms remained unmapped.
[b]Pagen et al. reported following symptoms (n=44): loss/change of smell; fatigue; shortness of breath; concentration difficulties; loss/change of taste; amnesia; chest pressure; hair loss; headache; brain fog; pain between shoulder blades; palpitations; increased resting heart rate; confusion; pain or burning sensation in the lungs; irritability; muscle pain or weakness; voice difficulties; diarrhea; elevated body temperature; heat flushes; fever; sudden weight loss; burning sensation in the trachea; stomach ache; dizziness; sleeping problems; loss of appetite; tinnitus; skin rash/red spots on toes or feet; nerve pain; vomiting; fear; eye difficulties; nausea; joint pain; coughing up mucus; sore throat; earache; coughing; dreariness/depression; sneezing; cold; runny nose.[2] In addition to the matches from the table (n=27), we also mapped brain fog, confusion, voice difficulties, loss of appetite, tinnitus, skin rash/red spots on toes or feet, eye difficulties, and dreariness/depression from "other" free-text symptoms (n=8). Unmapped symptoms (n=10): amnesia; pain or burning sensation in the lungs; irritability; heat flushes; sudden weight loss; burning sensation in the trachea; fear; earache; sneezing; cold.
[c]Sudre et al. reported following symptoms (n=14): delirium; abdominal pain; hoarse voice; diarrhea; chest pain; skipped meals; unusual muscle pains; fever; sore throat; persistent cough; loss of smell; shortness of breath; headache; fatigue.[3] In addition to the matches from the table (n=13), we also mapped hoarse voice from the "other" free-text symptoms (n=1). Unmapped symptoms (n=2): delirium; skipped meals.
[d]Thaweethai et al. reported following symptoms (n=12): PEM; fatigue; brain fog; dizziness; GI symptoms; palpitations; changes in sexual desire or capacity; loss of or change in smell or taste; thirst; chronic cough; chest pain; abnormal movements.[4] In addition to the matches from the table (n=12), we also mapped abnormal movements, brain fog, and GI symptoms from the "other" free-text symptoms (n=3). Unmapped symptoms (n=3): PEM; changes in sexual desire or capacity; thirst.
[e]Yoo et al. reported following symptoms (n=9): fatigue; shortness of breath; muscle aches; loss of smell or taste; chest pain; numbness or tingling; nausea, vomiting, or diarrhea; fever, chills, or night sweats; rash.[5] In addition to the matches above (n=12), we also mapped numbness or tingling, night sweats, and rash from the "other" free-text symptoms (n=3). No symptoms remained unmapped.
27 symptoms that were assessed in our INSPIRE cohort (22 from the INSPIRE questionnaire and 5 from the free-text “other” responses) appeared at least in one other set of symptoms in the comparator papers. 5 symptoms out of the 29 from the INSPIRE questionnaire (21 COVID-like symptoms and 8 fatigue symptoms) appeared in each of the five definitions (“more tired than usual”, “pain or tightness in your chest” , “Diarrhea (>3 loose/looser than normal stools/24 hours)”, “decreased smell or change in smell”, and “fatigue, tiredness, or exhaustion”).

© 2025 Wisk LE et al. *JAMA Network Open*.

## eTable 5. Comparison of Long COVID prevalence from the studies with INSPIRE COVID cohorts

| | | INSPIRE Long COVID Prevalence (%) | | | | | |
|---|---|---|---|---|---|---|---|
| | | 3-month Follow-up | | | 6-month Follow-up | | |
| **Study (Timepoint)** | **Reference Prevalence** | **COVID+ (N=3521)** | **COVID- (N=1054)** | **p-value** | **COVID+ (N=2986)** | **COVID- (N=911)** | **p-value** |
| Jones et al.,[1] 2021 (≥4 weeks) | 9.8 | 34.54 (32.97 - 36.14) | 31.69 (28.90 - 34.61) | 0.09 | 16.98 (15.66 - 18.39) | 16.68 (14.35 - 19.30) | 0.88 |
| Pagen et al.,[2] 2023 (Definition 6: 3-5 months, 6-11 months, ≥12 months) | 47.4, 61.9, 64.1 | 42.01 (40.37 - 43.66) | 40.32 (37.35 - 43.36) | 0.35 | 21.94 (20.47 - 23.47) | 23.27 (20.59 - 26.18) | 0.42 |
| Sudre et al.,[3] 2021 (≥28, ≥56, ≥84 days) | 13.3, 4.5, 2.6 | 35.1 (33.53 - 36.71) | 32.83 (30.01 - 35.77) | 0.18 | 16.85 (15.53 - 18.25) | 17.12 (14.77 - 19.76) | 0.88 |
| Thaweethai et al.,[4] 2023 (6 months) | 10.0 | 30.84 (29.33 - 32.40) | 28.08 (25.41 - 30.92) | 0.09 | 14.23 (13.01 - 15.55) | 14.599 (12.40 - 17.10) | 0.82 |
| Yoo et al.,[5] 2022 (≥ 60 days) | 29.8 | 31.64 (30.11 - 33.21) | 29.22 (26.51 - 32.09) | 0.15 | 15.3 (14.04 - 16.66) | 15.81 (13.53 - 18.38) | 0.75 |

**Notes:** Long COVID definition from each paper was applied to eligible INSPIRE participants who completed the surveys at 3-months and/or 6-months. INSPIRE cohort eligible for 3-month follow-up survey had n=4575 and for both 3- and 6-month survey, had n=3897 participants, respectively.
The prevalences were calculated based on persistence of ≥ 1 symptoms (constituting source definitions) at baseline and 3-month (for 3-month prevalence); and at baseline, 3-month, 6-month (for 6-month prevalence).
Prevalence (in %) are shown for the respective COVID cohort at each point of follow-up. 95% confidence intervals are shown in the parentheses.
The corresponding prevalences of Long COVID patients were tested for equality at 3-month (with participants completing baseline and 3-month surveys) and 6-month (with participants completing baseline, 3-month, and 6-month surveys) follow-up periods and p-values were provided.

© 2025 Wisk LE et al. *JAMA Network Open*.

## eTable 6. Symptom count by Long COVID definition

| Study | Long COVID status by study definition | 3-month | | | | 6-month | | | |
|---|---|---|---|---|---|---|---|---|---|
| | | Mean | SD | p-value | Median | Mean | SD | p-value | Median |
| Jones et al.,[1] 2021 | COVID+, with long COVID | 2.84 | 2.7 | 0.0006 | 2 | 1.87 | 1.8 | 0.02 | 1 |
| | COVID-, with long COVID | 2.27 | 2.7 | | 1 | 1.46 | 2 | | 1 |
| | COVID+, without long COVID | 0.06 | 0.3 | 0.98 | 0 | 0.02 | 0.2 | 0.65 | 0 |
| | COVID-, without long COVID | 0.06 | 0.4 | | 0 | 0.03 | 0.2 | | 0 |
| Pagen et al.,[2] 2023 | COVID+, with long COVID | 2.43 | 2.6 | 0.0001 | 2 | 1.53 | 1.8 | 0.005 | 1 |
| | COVID-, with long COVID | 1.88 | 2.6 | | 1 | 1.14 | 1.8 | | 1 |
| | COVID+, without long COVID | 0 | 0.1 | 0.87 | 0 | 0 | 0 | 0.58 | 0 |
| | COVID-, without long COVID | 0 | 0 | | 0 | 0 | 0 | | 0 |
| Sudre et al.,[3] 2021 | COVID+, with long COVID | 2.82 | 2.7 | 0.0006 | 2 | 1.88 | 1.8 | 0.02 | 1 |
| | COVID-, with long COVID | 2.26 | 2.7 | | 1 | 1.47 | 2 | | 1 |
| | COVID+, without long COVID | 0.05 | 0.2 | 0.02 | 0 | 0.02 | 0.2 | 0.20 | 0 |
| | COVID-, without long COVID | 0.03 | 0.2 | | 0 | 0.01 | 0.1 | | 0 |
| Thaweethai et al.,[4] 2023 | COVID+, with long COVID | 2.98 | 2.8 | 0.0009 | 2 | 1.98 | 2 | 0.01 | 1 |
| | COVID-, with long COVID | 2.37 | 2.9 | | 1 | 1.47 | 2.1 | | 1 |
| | COVID+, without long COVID | 0.14 | 0.5 | 0.49 | 0 | 0.06 | 0.3 | 0.70 | 0 |
| | COVID-, without long COVID | 0.13 | 0.5 | | 0 | 0.06 | 0.3 | | 0 |
| Yoo et al.,[5] 2022 | COVID+, with long COVID | 2.96 | 2.8 | 0.0008 | 2 | 1.96 | 1.9 | 0.01 | 1 |
| | COVID-, with long COVID | 2.36 | 2.8 | | 1.5 | 1.48 | 2.1 | | 1 |
| | COVID+, without long COVID | 0.12 | 0.4 | 0.18 | 0 | 0.04 | 0.2 | 0.46 | 0 |
| | COVID-, without long COVID | 0.1 | 0.4 | | 0 | 0.04 | 0.2 | | 0 |

**Notes:** Symptom count reflects the number of persistent symptoms of our 21 possible COVID symptoms assessed in the CDC screener. P-values were calculated based on t-test comparing the means of COVID+ vs COVID- groups assuming equal variance.

© 2025 Wisk LE et al. *JAMA Network Open*.

**eFigure 3. Mean symptom count by Long COVID definition at 3 months and 6 months**

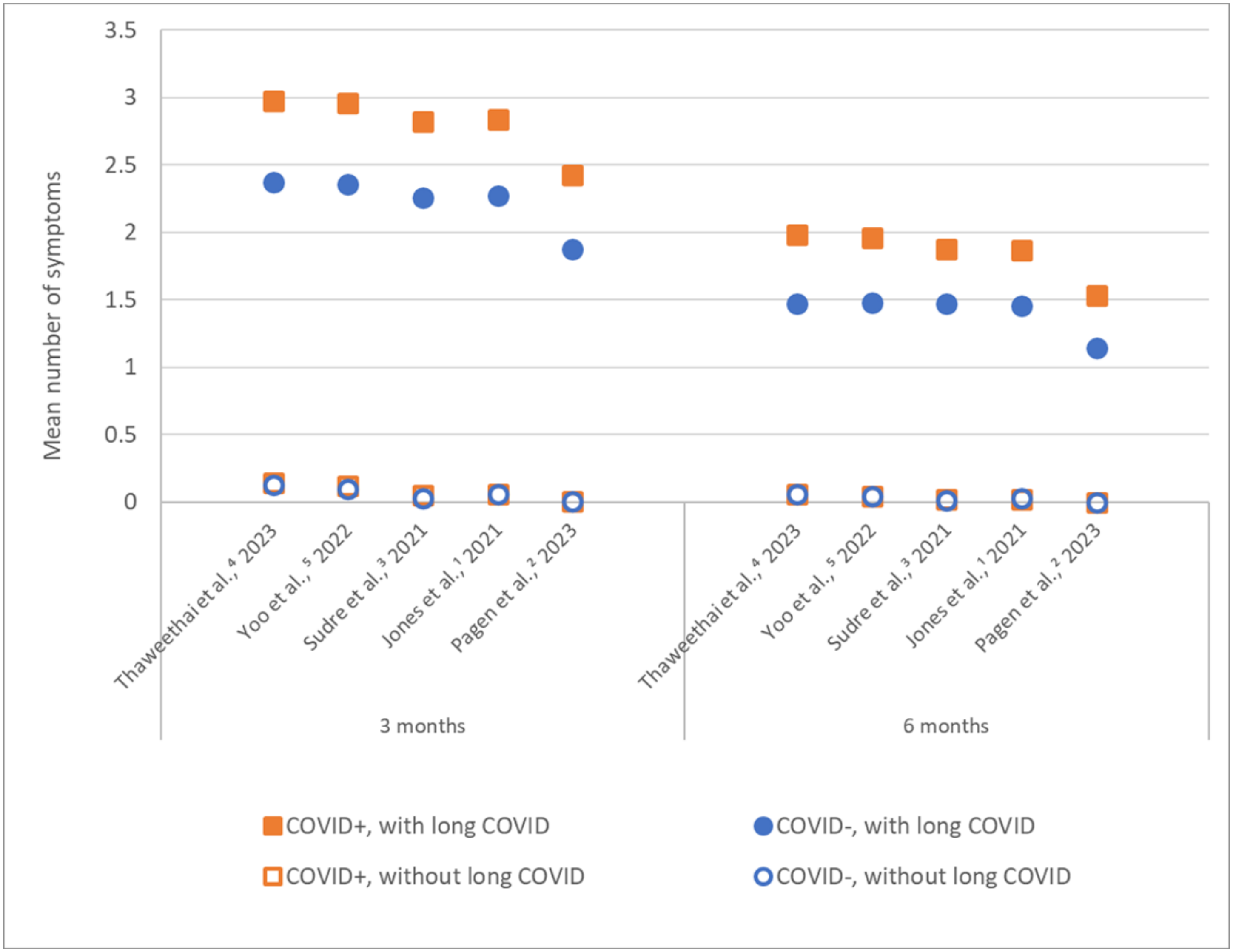


© 2025 Wisk LE et al. *JAMA Network Open*.

**eTable 7. 2x2 Tables comparing Long COVID counts from the INSPIRE final survey (participant self-report) vs. counts after applying the Long COVID from 5 studies**

| Source Paper | Index COVID status | INSPIRE Final Survey Self-reported Response | | | | | | | |
|---|---|---|---|---|---|---|---|---|---|
| | | COVID+ | | | | COVID- | | | |
| | | 3-month Follow-up | | 6-month Follow-up | | 3-month Follow-up | | 6-month Follow-up | |
| | | Positive | Negative | Positive | Negative | Positive | Negative | Positive | Negative |
| **Jones et al.,[1] 2021** | Positive | 396 | 354 | 236 | 107 | 77 | 129 | 42 | 54 |
| | Negative | 272 | 1287 | 368 | 1426 | 85 | 400 | 108 | 438 |
| **Pagen et al.,[2] 2023** | Positive | 443 | 483 | 275 | 176 | 96 | 169 | 52 | 79 |
| | Negative | 225 | 1158 | 329 | 1357 | 66 | 360 | 98 | 413 |
| **Sudre et al.,[3] 2021** | Positive | 395 | 373 | 236 | 106 | 77 | 138 | 42 | 57 |
| | Negative | 273 | 1268 | 368 | 1427 | 85 | 391 | 108 | 435 |
| **Thaweethai et al.,[4] 2023** | Positive | 361 | 311 | 192 | 88 | 66 | 116 | 36 | 47 |
| | Negative | 307 | 1330 | 412 | 1445 | 96 | 413 | 114 | 445 |
| **Yoo et al.,[5] 2022** | Positive | 371 | 307 | 213 | 93 | 73 | 118 | 40 | 52 |
| | Negative | 297 | 1334 | 391 | 1440 | 89 | 411 | 110 | 440 |

**Note:** Self-reported Long COVID status was obtained from the participants at the final INSPIRE survey

© 2025 Wisk LE et al. *JAMA Network Open*.

**eReferences**

1. Jones R, Davis A, Stanley B, et al. Risk Predictors and Symptom Features of Long COVID Within a Broad Primary Care Patient Population Including Both Tested and Untested Patients. *Pragmat Obs Res*. 2021;12:93-104. doi:10.2147/POR.S316186
2. Pagen DME, van Bilsen CJA, Brinkhues S, et al. Prevalence of Long-term Symptoms Varies When Using Different Post-COVID-19 Definitions in Positively and Negatively Tested Adults: The PRIME Post-COVID Study. *Open Forum Infect Dis*. 2023;10(10):ofad471. doi:10.1093/ofid/ofad471
3. Sudre CH, Murray B, Varsavsky T, et al. Attributes and predictors of long COVID. *Nat Med*. 2021;27(4):626-631. doi:10.1038/s41591-021-01292-y
4. Thaweethai T, Jolley SE, Karlson EW, et al. Development of a Definition of Postacute Sequelae of SARS-CoV-2 Infection. *JAMA*. 2023;329(22):1934-1946. doi:10.1001/jama.2023.8823
5. Yoo SM, Liu TC, Motwani Y, et al. Factors Associated with Post-Acute Sequelae of SARS-CoV-2 (PASC) After Diagnosis of Symptomatic COVID-19 in the Inpatient and Outpatient Setting in a Diverse Cohort. *J Gen Intern Med*. 2022;37(8):1988-1995. doi:10.1007/s11606-022-07523-3
6. Augustin M, Schommers P, Stecher M, et al. Post-COVID syndrome in non-hospitalised patients with COVID-19: a longitudinal prospective cohort study. *Lancet Reg Health Eur*. 2021;6:100122. doi:10.1016/j.lanepe.2021.100122
7. Carvalho-Schneider C, Laurent E, Lemaignen A, et al. Follow-up of adults with noncritical COVID-19 two months after symptom onset. *Clin Microbiol Infect*. 2021;27(2):258-263. doi:10.1016/j.cmi.2020.09.052

© 2025 Wisk LE et al. *JAMA Network Open*.

8. Desgranges F, Tadini E, Munting A, et al. Post-COVID-19 Syndrome in Outpatients: a Cohort Study. *J Gen Intern Med*. 2022;37(8):1943-1952. doi:10.1007/s11606-021-07242-1

9. Kisiel MA, Janols H, Nordqvist T, et al. Predictors of post-COVID-19 and the impact of persistent symptoms in non-hospitalized patients 12 months after COVID-19, with a focus on work ability. *Ups J Med Sci*. 2022;127. doi:10.48101/ujms.v127.8794

10. Klein H, Asseo K, Karni N, et al. Onset, duration and unresolved symptoms, including smell and taste changes, in mild COVID-19 infection: a cohort study in Israeli patients. *Clin Microbiol Infect*. 2021;27(5):769-774. doi:10.1016/j.cmi.2021.02.008

11. Lapa J, Rosa D, Mendes JPL, Deusdará R, Romero GAS. Prevalence and Associated Factors of Post-COVID-19 Syndrome in a Brazilian Cohort after 3 and 6 Months of Hospital Discharge. *Int J Environ Res Public Health*. 2023;20(1):848. doi:10.3390/ijerph20010848

12. Morioka S, Tsuzuki S, Suzuki M, et al. Post COVID-19 condition of the Omicron variant of SARS-CoV-2. *J Infect Chemother*. 2022;28(11):1546-1551. doi:10.1016/j.jiac.2022.08.007

13. Peghin M, Palese A, Venturini M, et al. Post-COVID-19 symptoms 6 months after acute infection among hospitalized and non-hospitalized patients. *Clin Microbiol Infect*. 2021;27(10):1507-1513. doi:10.1016/j.cmi.2021.05.033

14. Román-Montes CM, Flores-Soto Y, Guaracha-Basañez GA, et al. Post-COVID-19 syndrome and quality of life impairment in severe COVID-19 Mexican patients. *Front Public Health*. 2023;11:1155951. doi:10.3389/fpubh.2023.1155951

© 2025 Wisk LE et al. *JAMA Network Open*.
