# Supplementary material for: Variability in Long COVID Definitions and Validation of Published Prevalence Rates
Source: JAMA Netw Open. 2025 Aug 12;8(8):e2526506. doi: 10.1001/jamanetworkopen.2025.26506 (PMC12344537; doi:10.1001/jamanetworkopen.2025.26506)
Supplement: Supplement 3. — Data Sharing Statement [file jamanetwopen-e2526506-s003.pdf]

Data Sharing Statement

Wisk. Variability in Long COVID Definitions and Validation of Published Prevalence Rates. *JAMA Netw Open*. Published August 12, 2025. doi:10.1001/jamanetworkopen.2025.26506

Data
Data available: No
